# The Association Between Short-Term Blood Pressure Variability and Inflammation in Healthy Young Adults

**DOI:** 10.3390/jcdd12100399

**Published:** 2025-10-09

**Authors:** Charles J. Weeks, Bayu B. Bekele, Michelle Altvater, Jie Cheng, Haidong Zhu, Ying Huang, Deborah A. Jehu, Abigayle B. Simon, Wenjun Li, Yanbin Dong

**Affiliations:** 1 Medical College of Georgia, Augusta University, Augusta, GA 30912, USA; chweeks@augusta.edu (C.J.W.); absimon@augusta.edu (A.B.S.); 2 Georgia Prevention Institute, Augusta University, Augusta, GA 30912, USA; bbekele@augusta.edu (B.B.B.); hzhu@augusta.edu (H.Z.); yihuang@augusta.edu (Y.H.); wenjun_li@uml.edu (W.L.); 3 Department of Public Health, Center for Health Statistics and Biostatistics Core, University of Massachusetts-Lowell, Lowell, MA 01854, USA; jie_cheng@uml.edu; 4 Department of Community & Behavioral Health Sciences, School of Public Health, Augusta University, Augusta, GA 30904, USA; djehu@augusta.edu

**Keywords:** ambulatory blood pressure monitoring, blood pressure variability, cardiovascular diseases, cytokines, C-reactive protein, diastolic blood pressure, inflammation, tumor necrosis factor alpha, young adult

## Abstract

Blood pressure variability (BPV) is linked to cardiovascular disease (CVD) and systemic inflammation in adults, but its relevance in young, healthy populations remains unclear. This study examined the association between short-term BPV and inflammatory markers in 447 normotensive participants (mean age, 22.9 years) from the Georgia Stress and Heart (GSH) study, a cohort of Non-Hispanic Black and White individuals. Participants underwent 24 h ambulatory blood pressure monitoring and assessment of serum inflammatory markers, including hs-CRP, IFN-γ, IL-6, and TNF-α. BPV was quantified using average real variability (ARV), and generalized estimating equations (GEEs) were used to evaluate associations, adjusting for age, sex, race, and mean blood pressure. Diastolic BPV was significantly, positively associated with hs-CRP and TNF-α, whereas systolic BPV was not associated with any inflammatory marker. Specifically, 24 h diastolic BPV was positively associated with hs-CRP (*p* = 0.001) and TNF-α (*p* = 0.015), while daytime diastolic BPV was positively associated with hs-CRP (*p* = 0.002). Nighttime diastolic BPV was positively associated with both hs-CRP (*p* = 0.020) and TNF-α (*p* = 0.007). No significant associations were found between BPV and IL-6 or IFN-γ. These findings suggest diastolic BPV may be a marker of low-grade inflammation in healthy young adults and could represent an early cardiovascular risk factor that warrants longitudinal study.

## 1. Introduction

Although hypertension (HTN) is the strongest predictor of cardiovascular disease (CVD) [1] and inflammatory cytokine concentration can predict future HTN [2], blood pressure variability (BPV) metrics have been associated with CVD and inflammation in various forms [3,4,5,6,7,8,9]. The precise mechanism of action by which this occurs is not well elucidated. This variability can be short-term or long-term. Short-term BPV has various forms, but it most prominently represents ambulatory variability over a period of up to 24 h while long-term BPV includes variability over weeks, months, seasons, and even years [9].

Over recent years, mounting evidence has underscored the pivotal role of long-term BPV as an independent predictor of adverse cardiovascular, hypoperfusion outcomes, and mortality [10,11,12,13,14,15]. Meanwhile inflammatory markers such as C-reactive protein (CRP) [16], interleukin-6 (IL-6) [17,18], and tumor necrosis factor alpha (TNF-α) [19,20] are all independently associated with increased risk for CVD and mortality. Notably, elevated levels of IL-6 [7] and high-sensitivity C-reactive protein (hs-CRP) [21] have been implicated in increased long-term BPV, indicating a potential role of systemic inflammation in exacerbating BP fluctuations and amplifying CVD risk.

In addition to prior work on BPV and inflammation, studies have highlighted the broader complexity of cardio-immune interactions. Multiplex profiling of cytokines improved risk stratification for adverse outcomes [22], while other studies described the role of neuroimmune crosstalk in the pathophysiology of hypertension [23]. For example, IL-17 is a pro-inflammatory cytokine and has been implicated in hypertension through vascular inflammation and endothelial dysfunction with reduced nitric oxide bioavailability, further supporting the biological plausibility of BPV as an immune-linked phenotype [24,25].

Short-term BPV also independently predicts end-organ damage in hypertensive adults [26], and BPV is known to be greater in hypertensive than in normotensive adults [27,28]. Systolic short-term BPV exhibits pronounced prognostic implications for mortality in young adults [29], and prior studies have demonstrated an association between short-term BPV and kidney damage [30,31,32]. However, meta-analysis showed that although higher diastolic and systolic BPV predicted total and cardiovascular mortality, mean blood pressure (BP) was the primary risk factor [5].

Short-term BPV has also been associated with inflammation. Studies have revealed associations between inflammatory markers, such as CRP, IL-6, and TNF-α with BPV in older hypertensive patients, suggesting a potential mechanistic link between inflammation and target organ damage in hypertension [33]. In young people, IL-6 and CRP are important as indicators of low-grade systemic inflammation, which has been associated with both mental [34,35,36] and cardiometabolic health risks [37,38,39]. However, to the best of our knowledge, no studies have evaluated a possible association between short-term BPV in young healthy patients and serum inflammatory markers. We hypothesize that there would be a direct relationship.

## 2. Materials and Methods

### 2.1. Study Population/Trial Design

The participants were from the Georgia Stress and Heart (GSH) study. This investigation represents a post hoc analysis of data collected during visits 13–15 of the GSH study. Inclusion criteria included (1) Non-Hispanic Blacks or Non-Hispanic White, (2) aged 5 to 16 in 1989, (3) normotensive at the time of BP screening, (4) apparently healthy. Participants were considered ‘apparently healthy’ if they were normotensive and free of chronic disease or acute illness at the time of screening, as determined by standardized health history and physical examination. Participants were screened for eligibility and recruited from the region around Augusta, Georgia. Parents completed a family health history questionnaire at the public school screening for students in kindergarten through 8th grade. The original cohort consisted of 740 participants and began rolling enrollment in 1989. The GSH study entailed multiple visits over time where the participants were evaluated for various factors. On three of these visits (visits 13–15), participants had anthropometry measurements and a blood draw for inflammatory markers and then wore an ambulatory blood pressure monitor (ABPM) for 24 h. This study consisted of the 447 participants who attended at least one of these three visits. Participants with two visits had a mean interval of 20.7 months between visits, while those with three visits averaged 18.4 months. Therefore, 293 individuals from the original cohort were either lost to follow up, were unable to wear the ABPM for 24 h, or stopped participating in the study before these visits. Inflammatory markers were used in analysis when available. The Institutional Review Board at the Augusta University approved the present study as exempt from IRB review (1385407-3, 21 May 2019, exemption category #4). Informed consent was provided by all participants or parents if participants were <18 years in the original cohort.

### 2.2. Anthropometry Measurements

Height was measured to the nearest 0.1 cm by a wall-mounted stadiometer (TanitaCorporation of American, Arlington Heights, IL, USA); weight was measured to the nearest 0.1 kg by a calibrated electronic scale with the participants not wearing shoes and in light clothing (model CN2OL; Cardinal Detecto, Webb City, MO, USA). Waist circumference was measured at the center of the umbilicus. Hip circumference was measured at the widest part of the hips. Waist-to-hip ratio (WHR) was computed as the waist circumference divided by the hip circumference.

### 2.3. Blood Pressure Variability

BPV was evaluated by the metrics obtained by the ABPM (model 90207; SpaceLabs, Redmond, WA, USA) that was fitted to the nondominant arm. Readings were recorded every 20 min during the daytime (8 AM to 10 PM) and every 30 min during the nighttime (12 AM to 6 AM). Transitional periods from 6 AM to 8 AM h and 10 PM to 12 AM were not included in the analyses. Adequacy of recordings were based on acceptable readings using previously established criteria [40,41]. We required at least 14 readings over the 14 h designated as daytime and at least six readings over the 6 h designated as the nighttime for inclusion. The weighted 20 h BPV used in this study is the mean of the daytime BPV and nighttime BPV weighted for the duration of daytime and nighttime subperiods in the same fashion that has previously been carried out for this cohort [42]. We considered this weighted 20 h BPV to represent 24 h BPV for consistency in reporting with the current literature. ARV was used as the metric for BPV.

(1) $$ARV=\left[\frac{1}{n\;-\;1}\sum_{i\;=\;1}^{n\;-\;1}\left|{BP}_{i\;+\;1}\;-\;{BP}_{i}\right|\right]$$

ARV has been shown to be the best model for assessing 24 h BPV [43,44].

### 2.4. Plasma Inflammatory Marker Measurement

hs-CRP, IFN-γ, IL-6, and TNF-α were measured using a Simple Plex assay, which was based on microfluidics and glass nanoreactor (GNR) technology (Simple Plex, Protein Simple Corp., San Jose, CA, USA) [45]. The Ella platform automates the immunoassay by running samples in parallel through 158 individual microfluidic channels, binding the protein of interest before washing off unbound 159 analyte and adding a detection reagent. Each channel has three GNRs that are coated with a 160-capture antibody so that results are produced in triplicate for each sample. The intra-assay and inter-assay CVs were all <7.0%.

### 2.5. Statistical Analysis

Continuous variables were summarized using means and standard deviations (mean ± SD) and compared across number of visits group using one-way analysis of variance (ANOVA). Categorical variables were presented as frequencies and percentages, and group comparisons were performed using Pearson’s chi-squared test. Subjects were analyzed for baseline demographics, BPV, and inflammatory markers. Four continuous dependent variables (hs-CRP, IFNg, IL-6, and TNF-α) were log10-transformed due to their skewed distribution to the right. ARV was used to measure BPV as the primary independent variable. The associations between inflammatory markers and BPV were analyzed using generalized estimating equations (GEEs) with an unstructured correlation structure to account for the correlation between repeated measurements within subjects. GEEs account for within-subject correlation across visits rather than averaging values. Models were adjusted for potential confounders, including age, sex, race, and mean blood pressure. The quasi-likelihood under the independence model criterion (QIC) was used to select the best-fitting model. Because this was a post hoc analysis of an existing cohort, no a priori sample size calculation was performed. Each inflammatory marker that was significantly associated with BPV in the adjusted GEE analysis was also modeled with an unadjusted simple linear regression analysis for figure production. Bivariate correlations were used for the associations between inflammatory markers. All statistical analyses were performed using Stata Statistical Software (StataCorp. 2023. Release 18. College Station, TX, USA: StataCorp LLC), and figures were produced with GraphPad Prism Version 10. A two-tailed *p*-value < 0.05 was considered statistically significant.

## 3. Results

### 3.1. Baseline Characteristics

The mean age of participants was 22.90 ± 3.11 years across all participants (*n* = 447), but there was variation among subgroups (*p* < 0.01) at baseline. Participants with one visit (*n* = 144) had a mean age of 23.63 ± 3.21 years, those with two visits (*n* = 210) averaged 23.69 ± 3.19 years, and those with three visits (*n* = 93) averaged 21.34 ± 2.21 years old at baseline. Subjects who came to two visits had 20.66 months on average between visits (95% CI 19.85–21.46), and subjects who attended three visits had 18.39 months between visits on average (95% CI 17.98–18.81). In terms of sex distribution, the cohort consisted of 212 males (47.43%) and 235 females (52.57%). Among participants with one visit, 46.53% were male (*n* = 67) and 53.47% were female (*n* = 77). Similarly, participants with two visits included 104 males (49.52%) and 106 females (50.48%), while the three-visit group consisted of 41 males (44.09%) and 52 females (55.91%). Regarding race, 210 participants (46.98%) identified as Non-Hispanic White, and 237 (53.02%) identified as Non-Hispanic Black (Table 1).

The mean systolic ABP was 118.44 ± 9.62 mmHg, and the mean diastolic ABP was 70.74 ± 7.50 mmHg. The mean systolic ABP (*p* < 0.01) and mean diastolic ABP (*p* < 0.01) were significantly different among groups of participants who attended one, two, or three visits. However, variability in ABP was not significantly different among groups for systolic or diastolic pressures over any time period. The mean 24 h systolic BPV was 7.75 ± 1.41, with daytime and nighttime values of 7.79 ± 1.65 and 7.80 ± 2.52, respectively. The mean 24 h diastolic BPV was 7.62 ± 1.50, with daytime and nighttime values of 7.79 ± 1.77 and 7.49 ± 2.54, respectively (Table 1).

Serum cytokine concentrations were collected in available participants across the cohort and were not significantly different between groups. The mean hs-CRP concentration was 3.39 × 10^−2^ ± 7.25 × 10^−2^ mg/L. IL-6 concentrations were 3.83 ± 7.38 pg/mL, TNF-α had a mean concentration of 6.30 ± 5.39 pg/mL, and IFN-γ averaged 0.87 ± 1.17 pg/mL (Table 1).

### 3.2. Associations Between BPV and Inflammation

**Figure 1:**
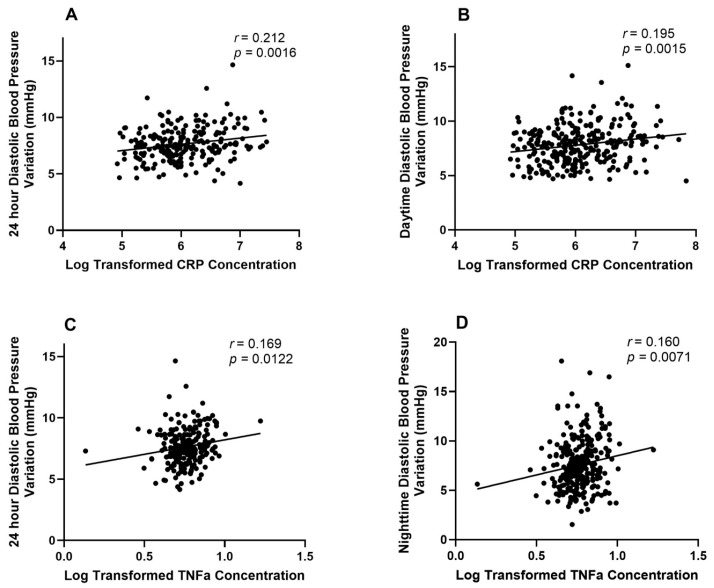
Linear regression models of the significant inflammatory markers and blood pressure variation associations. (**A**) The association between CRP and 24 h diastolic BPV, (**B**) the association between CRP and daytime diastolic BPV, (**C**) the association between CRP and 24 h diastolic BPV, and (**D**) the association between CRP and 24 h diastolic BPV.

For hs-CRP, the 24 h systolic BPV had a positive but non-significant association (estimate = 0.048, standard error = 0.030, 95% CI: −0.010 to 0.107, *p* = 0.104). Daytime and nighttime systolic BPV showed non-significant associations (daytime: 0.017, *p* = 0.364; nighttime: 0.019, *p* = 0.136) with hs-CRP. In contrast, the diastolic BPV measures revealed more robust associations with hs-CRP. The 24 h diastolic BPV exhibited a significant positive relationship (estimate = 0.076, standard error = 0.023, 95% CI: 0.031 to 0.120, *p* = 0.001). Additionally, the daytime diastolic BPV was significantly associated with hs-CRP (estimate = 0.054, standard error = 0.017, 95% CI: 0.020 to 0.088, *p* = 0.002) (Table 2 and Figure 1). This was also true for the nighttime diastolic BPV (estimate = 0.028, standard error = 0.012, 95% CI: 0.005 to 0.052, *p* = 0.020).

Diastolic BPV measures were significantly associated with elevated TNF-α levels. The 24 h diastolic BPV showed a positive relationship (estimate = 0.011, standard error = 0.005, 95% CI: 0.002 to 0.021, *p* = 0.015), and the nighttime diastolic BPV also reached significance (estimate = 0.006, *p* = 0.007). Systolic BPV measures did not show associations with TNF-α, with *p*-values ranging from 0.470 to 0.954 (Table 2 and Figure 1).

For IFN-γ, no significant associations were observed. The 24 h systolic BPV had a minimal estimate of 0.002 (*p* = 0.886), and the nighttime systolic BPV showed a slightly negative but non-significant association (estimate = −0.005, *p* = 0.655). Diastolic BPV measures, including the 24 h BPV (estimate = −0.003, *p* = 0.765) and daytime BPV (estimate = −0.006, *p* = 0.605), also did not exhibit meaningful relationships. No significant relationships were found across BPV measures for IL-6. The 24 h systolic BPV had a small estimate of 0.008 (*p* = 0.546), and the 24 h diastolic BPV had a non-significant positive estimate of 0.018 (*p* = 0.110). Other systolic and diastolic BPV measures similarly failed to show meaningful associations (Table 2).

Correlation analysis demonstrated significant positive associations between hs-CRP and IL-6 (*r* = 0.434, *p* < 0.001) and between hs-CRP and TNF-α (*r* = 0.292, *p* < 0.001). IL-6 was also significantly correlated with TNF-α (*r* = 0.369, *p* < 0.001). No significant correlations were observed between IFN-γ and any of the other inflammatory markers (Supplemental Table S1).

## 4. Discussion

This study examined the relationship between short-term BPV and systemic inflammation. The findings highlight the association of diastolic BPV with inflammatory biomarkers, particularly hs-CRP and TNF-α, but show limited associations with systolic BPV.

This is a unique cohort consisting of young adults with a mean age of approximately 23 years, a balanced sex distribution, and an even distribution of Non-Hispanic White and Black participants. Notably, the baseline BP values fell within the normotensive range, with systolic and diastolic mean values of 118 mmHg and 71 mmHg, respectively. These baseline characteristics establish that this population represents a relatively healthy young group, distinct from the older or hypertensive cohorts that typically dominate cardiovascular risk studies.

### 4.1. Diastolic BPV

The GEE analysis demonstrated that diastolic BPV, compared to systolic BPV, had stronger and more consistent associations with key inflammatory markers. Specifically, 24 h diastolic and nighttime BPV was significantly associated with both CRP and TNF-α. Daytime diastolic BPV also exhibited a significant positive association with CRP. This suggests that fluctuations in diastolic BP may coincide with systemic inflammation. These findings align with previous studies that showed increased diastolic BPV was associated with the development of cognitive decline, end-stage renal disease, hypertension, and adverse cardiovascular outcomes, many of which are driven in part by inflammatory pathways and endothelial dysfunction [5,46,47,48]. The persistence of associations after adjusting for mean BP suggests that variability itself, beyond average levels, may contribute to inflammation.

Importantly, associations emerged even in this young population where diastolic BPV typically increases with age. The repeated-measures design with intervals of ~18–21 months allowed us to account for within-subject changes while recognizing that aging-related changes may still occur even in this relatively young cohort. Aging leads to declining tissue and vascular function, reduced arterial compliance, and disrupted cardiovascular homeostasis, which is linked to worse outcomes like cardiovascular and all-cause mortality. Young adults typically have more flexible, adaptive BP control [49]. Although effect sizes were small, this is expected in a young, relatively healthy sample where systemic inflammation is low. Even modest associations may be biologically meaningful and could precede more pronounced changes later in life.

### 4.2. Systolic BPV

In contrast, systolic BPV measures—whether assessed over 24 h, during the day, or at night—were not significantly associated with any inflammatory markers. This finding suggests that short-term systolic fluctuations may have less correlation with inflammatory processes compared to diastolic variability. This may be due to differences in the physiological mechanisms governing systolic and diastolic pressures, with diastolic pressure reflecting peripheral vascular resistance and being more sensitive to autonomic regulation, stress, and inflammation [50].

### 4.3. Inflammatory Cytokines

Among the cytokines analyzed, hs-CRP and TNF-α demonstrated the most robust associations with BPV. Elevated CRP, a well-established marker of systemic inflammation, was significantly linked to both the 24 h and daytime diastolic BPV, indicating that greater fluctuations in diastolic pressure may drive low-grade inflammation even in normotensive populations. Similarly, TNF-α, a pro-inflammatory cytokine involved in chronic inflammation, was significantly associated with 24 h and nighttime diastolic BPV, highlighting the importance of diastolic variability during sleep periods. These findings align with the broader literature suggesting that nighttime BPV, in particular, is associated with adverse cognitive and cardiovascular outcomes and may reflect autonomic dysregulation [51,52,53].

In contrast, the remaining cytokines—IL-6 and IFN-γ—exhibited no significant associations with BPV. The reason that CRP and TNF-α were associated with BPV while IL-6 and IFN-γ were not, remains unclear. IL-6 contributes to hypertension by activating Janus kinase and signal transducer pathways, increasing sodium channel activity in kidney tubules, and promoting sodium and water retention, particularly in angiotensin II–driven models [49]. IFN-γ, produced by T helper type 1 cells, enhances renal angiotensinogen expression through Janus kinase and signal transducer pathways, linking immune responses to activation of the renin–angiotensin system and raising BP [54]. The associations between BPV and CRP, as well as TNF-α, were small, so the cohort may have been underpowered or had too little inflammation for there to be significant associations between BPV and IFN-γ or IL-6. The differential findings, where hs-CRP and TNF-α but not IL-6 or IFN-γ were associated with BPV, may reflect differences in cytokine biology, sensitivity to low-grade inflammation, or limited statistical power for less variable markers.

### 4.4. Limitations

This study has many strengths including its sample size, its unique population, and the fact that it showed an association between BPV and inflammatory cytokines in a healthy population far before CVD would be expected. However, it does have some limitations. First, unstructured GEE analysis limits the ability to establish causal relationships between BPV and inflammation. Second, the cohort consisted of relatively healthy, normotensive young adults, which may limit comparability to studies on older or hypertensive populations where BPV and inflammation may behave differently. Third, inflammatory markers were not repeatedly measured throughout the day, preventing assessment of temporal fluctuations in inflammation relative to BPV. Finally, physical activity and stress were not recorded, which can affect both inflammatory marker concentration and BPV. Multiple testing across markers also raises the possibility of type I error, although the consistency of diastolic associations strengthens confidence in our findings. Our study lacked diseased controls, concurrent glucose and cholesterol measures, and detailed lifestyle data (e.g., exercise, smoking, and diet), which may confound associations with inflammation. Additionally, no a priori power calculation was performed given the post hoc design.

### 4.5. Implications

These findings have important clinical implications. While the study population did not exhibit elevated mean BP values, the associations between diastolic BPV and inflammation suggest that even in ostensibly healthy populations, variability in BP may influence systemic inflammation. This highlights the potential utility of monitoring diastolic BPV, especially during sleep and over extended periods, to identify individuals at higher risk for developing chronic inflammatory or cardiovascular conditions. More broadly, these results suggest that BP variability may represent a novel cardiovascular risk factor in young people, complementing traditional risk markers such as glucose, cholesterol, obesity, and smoking and warranting integration into prevention strategies.

### 4.6. Future Steps

The study also underscores the need for further investigation into the mechanisms linking diastolic BPV to inflammation. While the present findings suggest that diastolic variability is a stronger predictor of inflammatory markers than systolic variability, even in this young cohort, the underlying physiological processes remain unclear. Understanding these mechanisms could inform future targeted interventions to manage both BPV and inflammation in clinical practice. Also, longitudinal studies would be essential to determine whether reducing diastolic BPV through lifestyle or pharmacological interventions can mitigate inflammation and ultimately lower cardiovascular risk.

## 5. Conclusions

In conclusion, this study highlights the importance of short-term diastolic BPV as a significant correlate of systemic inflammation, particularly through its associations with CRP and TNF-α. These findings suggest that monitoring diastolic BPV, especially over 24 h and during nighttime periods, may provide valuable insights into the inflammatory status of individuals. Recognition of BPV as a novel cardiovascular risk factor in early adulthood has implications for both research and prevention, supporting its inclusion in the broader landscape of cardiovascular risk assessment.

## Figures and Tables

**Table 1:** Demographic, BPV and serum inflammatory descriptive results.

Characteristic	Visit 13–15 All Patients (*n* = 447)	1 Visit (*n* = 144)	2 Visits (*n* = 210)	3 Visits (*n* = 93)	*p*-Value
**Age, years**	22.90 ± 3.11	23.63 ± 3.21	23.69 ± 3.19	21.34 ± 2.21	<0.01 *
**Sex, *n* (%)**					
-Male	212 (47.43%)	67 (46.53%)	104 (49.52%)	41 (44.09%)	-
-Female	235 (52.57%)	77 (53.47%)	106 (50.48%)	52 (55.91%)	-
**Race, *n* (%)**					
Non-Hispanic White	210 (46.98%)	64 (44.44%)	92 (43.81%)	54 (58.06%)	-
Non-Hispanic Black	237 (53.02%)	80 (55.56%)	118 (56.19%)	39 (41.94%)	-
**Mean Systolic BP, mmHg**	118.44 ± 9.62	119.93 ± 11.19	118.95 ± 9.43	116.91 ± 8.83	<0.01 *
**Mean Diastolic BP, mmHg**	70.74 ± 7.50	72.19 ± 8.84	71.13 ± 7.67	69.40 ± 6.20	<0.01 *
**20 h BPV Systolic BP**	7.75 ± 1.41	7.87 ± 1.71	7.72 ± 1.28	7.74 ± 1.46	0.74
**Daytime BPV Systolic BP**	7.79 ± 1.65	7.91 ± 1.90	7.72 ± 1.59	7.85 ± 1.63	0.53
**Nighttime BPV Systolic BP**	7.80 ± 2.52	7.66 ± 2.43	8.02 ± 1.28	7.54 ± 2.48	0.08
**20 h BPV Diastolic BP**	7.62 ± 1.50	7.73 ± 1.60	7.65 ± 1.46	7.53 ± 1.51	0.59
**Daytime BPV Diastolic BP**	7.79 ± 1.77	7.84 ± 1.86	7.83 ± 1.75	7.71 ± 1.77	0.73
**Nighttime BPV Diastolic BP**	7.49 ± 2.54	7.55 ± 2.41	7.65 ± 2.17	7.23 ± 2.46	0.17
**CRP Serum Concentration mg/** **L**	3.39 × 10^−3^ ± 7.25 × 10^−3^	4.06 × 10^−3^ ± 1.06 × 10^−2^	3.51 × 10^−3^ ± 7.45 × 10^−3^	2.84 × 10^−3^ ± 4.15 × 10^−3^	0.54
**IL6 Serum Concentration pg/m** **L**	3.83 ± 7.38	5.75 ± 10.50	3.65 ± 7.90	2.97 ± 1.88	0.20
**TNF-α Serum Concentration pg/m** **L**	6.30 ± 5.39	6.10 ± 1.31	6.58 ± 7.27	5.94 ± 1.35	0.56
**IFN-γ Serum Concentration pg/m** **L**	0.87 ± 1.17	0.90 ± 1.28	0.94 ± 1.38	0.75 ± 0.55	0.46

Values are mean ± SD or n (%), * indicates a significance with a *p*-values < 0.05, *p*-values are from ANOVA or χ^2^; and BPV is reported as ARV units.

**Table 2:** Associations between serum inflammatory markers and BPV.

Variable	Estimate (β)	Standard Error	95% Confidence Interval	*p*-Value
**CRP**				
- Systolic 24 h BPV	0.048	0.03	−0.010, 0.107	0.104
- Systolic Day BPV	0.017	0.019	−0.020, 0.054	0.364
- Systolic Night BPV	0.019	0.013	−0.006, 0.045	0.136
- Diastolic 24 h BPV	0.076	0.023	0.031, 0.120	0.001 *
- Diastolic Day BPV	0.054	0.017	0.020, 0.088	0.002 *
- Diastolic Night BPV	0.028	0.012	0.005, 0.052	0.020 *
**IFN-γ**				
- Systolic 24 h BPV	0.002	0.016	−0.029, 0.034	0.886
- Systolic Day BPV	−0.005	0.013	−0.030, 0.020	0.677
- Systolic Night BPV	−0.003	0.007	−0.016, 0.010	0.655
- Diastolic 24 h BPV	−0.003	0.011	−0.025, 0.018	0.765
- Diastolic Day BPV	−0.006	0.011	−0.027, 0.016	0.605
- Diastolic Night BPV	−0.001	0.007	−0.013, 0.013	0.986
**TNF-α**				
- Systolic 24 h BPV	0.004	0.005	−0.006, 0.014	0.357
- Systolic Day BPV	0.000	0.004	−0.007, 0.006	0.747
- Systolic Night BPV	−0.001	0.002	−0.005, 0.003	0.443
- Diastolic 24 h BPV	0.011	0.005	0.002, 0.021	0.015 *
- Diastolic Day BPV	0.003	0.003	−0.003, 0.009	0.915
- Diastolic Night BPV	0.006	0.002	0.002, 0.010	0.007 *
**IL6**				
- Systolic 24 h BPV	0.008	0.013	−0.018, 0.035	0.546
- Systolic Day BPV	0.008	0.009	−0.009, 0.026	0.350
- Systolic Night BPV	−0.004	0.007	−0.017, 0.010	0.574
- Diastolic 24 h BPV	0.018	0.011	−0.004, 0.040	0.110
- Diastolic Day BPV	0.010	0.009	−0.009, 0.028	0.309
- Diastolic Night BPV	0.011	0.008	−0.004, 0.026	0.150

* indicates a significance with a *p*-values < 0.05 and *p*-values are from GEE analysis.

## Data Availability

The original contributions presented in this study are included in the article/Supplementary Material. Further inquiries can be directed to the corresponding author.

## Supplementary Materials

The following supporting information can be downloaded at: https://www.mdpi.com/article/10.3390/jcdd12100399/s1, Table S1: Correlations between inflammatory markers.

## Abbreviations

The following abbreviations are used in this manuscript:

BPV	Blood pressure variation
ABPM	Ambulatory blood pressure monitoring
ABP	Ambulatory blood pressure
CVD	Cardiovascular disease
CRP	C-reactive Protein
IL-6	Interleukin-6
TNF-α	Tumor necrosis factor alpha
GSH	Georgia Stress and Heart
ARV	Average Real Variability
GEE	Generalized Estimating Equations
QIC	Quasi-likelihood under the Independence model Criterion
IFN-γ	Interferon-gamma
SBP	Systolic blood pressure
DBP	Diastolic blood pressure
