# Supplementary material for: The Association Between Short-Term Blood Pressure Variability and Inflammation in Healthy Young Adults
Source: J Cardiovasc Dev Dis. 2025 Oct 9;12(10):399. doi: 10.3390/jcdd12100399 (PMC12564484; doi:10.3390/jcdd12100399)
Supplement: Supplementary file 1 [file jcdd-12-00399-s001.zip › jcdd-3872436-table-SI.pdf]

Supplemental Table 1. Correlations between inflammatory markers

**Variable 1**	**Variable 2**	***r***	***p***
CRP	IL-6	0.434	<0.001
CRP	TNF-α	0.292	<0.001
CRP	IFN-γ	0.032	0.330
IL-6	TNF-α	0.369	<0.001
IL-6	IFN-γ	0.030	0.467
TNF-α	IFN-γ	0.041	0.203
